# Assessment of Reliability and Validity of Celiac Disease–Related YouTube Videos: Content Analysis

**DOI:** 10.2196/58615

**Published:** 2025-02-04

**Authors:** Yunus Halil Polat, Rasim Eren Cankurtaran

**Affiliations:** 1 Department of Gastroenterology Ankara Training and Research Hospital Ankara Turkey; 2 Department of Gastroenterology Ankara Etlik City Hospital Ankara Turkey

**Keywords:** gastroenterology, celiac, YouTube, internet-based information, medical information, health-related, reliability, validity, quality, videos, celiac sprue, sprue, gluten enteropathy, cross-sectional

## Abstract

**Background:**

YouTube is an increasingly used platform for medical information. However, the reliability and validity of health-related information on celiac disease (CD) on YouTube have not been determined.

**Objective:**

This study aimed to analyze the reliability and validity of CD-related YouTube videos.

**Methods:**

On November 15, 2023, a search was performed on YouTube using the keyword “celiac disease.” This search resulted in a selection of videos, which were then reviewed by 2 separate evaluators for content, origin, and specific features. The evaluators assessed the reliability and quality of these videos using a modified DISCERN (mDISCERN) score, the *Journal of the American Medical Association* (*JAMA*) benchmark criteria score, the usefulness score, video power index (VPI), and the Global Quality Scale (GQS) score.

**Results:**

In the analysis of 120 initially screened CD videos, 85 met the criteria for inclusion in the study after certain videos were excluded based on predefined criteria. While the duration of the videos uploaded by health care professionals was significantly longer than the other group (*P*=.009), it was concluded that the median scores for mDISCERN (4, IQR 4-5 vs 2, IQR 2-3; *P*<.001), GQS (4, IQR 4-5 vs 3, IQR 2-3; *P*<.001), *JAMA* (4, IQR 3-4 vs 2, IQR 2-3; *P*<.001), and usefulness (8, IQR 7-9 vs 6, IQR 3-6; *P*<.001) of the videos from this group were significantly higher than those from non–health care professionals. Video interaction parameters, including the median number of views, views per day, likes, dislikes, comments, and VPI, demonstrated no significant difference between the 2 groups.

**Conclusions:**

This study showed that YouTube videos about CD vary significantly in reliability and quality depending on their source. Increasing the production of reliable videos by health care professionals may help to improve patient education and make YouTube a more reliable resource.

## Introduction

Celiac disease (CD) is an autoimmune disorder that occurs in genetically predisposed individuals as a result of the immune reaction to gluten, primarily affecting the small intestine [1]. Symptoms range from asymptomatic to digestive problems and nutritional deficiencies due to malabsorption of nutrients. Treatment includes a gluten-free diet [1]. Over the past few decades, CD has been estimated to affect around 1% of the world’s population [2]. Despite the increasing prevalence of CD, the majority of the patients with CD remain undiagnosed [1].

In recent years, the internet has become an important source of health information for the public. It has been reported that 80% of internet users use social media (SM) platforms to get information about their disease. Patients with chronic diseases in particular are increasingly relying on SM platforms to manage their conditions [3]. In a recent study investigating the use of SM by patients with CD and parents of patients with CD, it was reported that 96% of participants used SM for disease management [4]. YouTube (Google), is one of the world’s most popular video-sharing platforms. Currently, YouTube has more than 1 billion registered users, and billions of videos are watched every day, about 30 million of which are health-related. Health-related videos can be uploaded by anyone, but the content of these videos may contain inaccurate or misleading information without being reviewed by health care professionals.

There are studies in the literature evaluating the reliability and quality of YouTube videos for many diseases [5,6]. There are few studies evaluating CD-related YouTube videos [7,8]. However, one of these studies evaluated non-English language videos [8]. The other study did not measure CD-related YouTube videos with the tests developed for these studies and did not include videometric parameters (such as the number of likes and dislikes) in the evaluation [7]. Unlike previous studies, which either focused on non-English videos or lacked comprehensive quality metrics, this research provides a more robust and comparative analysis of CD-related video content on YouTube.

We could not find any studies in the literature that evaluated the reliability and validity of YouTube videos about CD. This study aims to evaluate the quality and reliability of YouTube videos about CD using validated scoring tools and detailed content analysis.

## Methods

### Study Design

In this cross-sectional study, videos were collected using the keyword “Celiac Disease” in YouTube’s search engine on November 15, 2023. The search was conducted in a Google Chrome browser in incognito mode, logged out of any user account, and using a standard IP address in Turkey. This was chosen because it is the most common keyword that holistically assesses all aspects of the disease, such as clinical, pathogenesis, diet, and nutrition. YouTube’s default relevance mode was used to simulate the average consumer’s search habits. It is recognized that most viewers rarely venture beyond the first few pages of results. Therefore, the first 120 videos about CD were selected, similar to previous studies. Based on the search results, a total of 120 videos were saved for further analysis, ranging from the most viewed video to the least viewed video. Video sampling criteria were determined with reference to similar studies [5,9].

The following factors were considered as exclusion criteria in the research: (1) videos in languages other than English, (2) videos with muted or poor picture quality, (3) videos containing advertisements, (4) videos with content unrelated to CD, and (5) videos with repetitive content.

### Data Review

Data such as video type (real and animation), video length (min), time since upload (d), number of views, number of daily views (number of views/d since upload), number of likes, number of daily likes (number of likes/d since upload), number of dislikes, and number of comments were recorded. In our study, we categorized video sources into two groups: educational content of health care professionals (doctors, academic institutions or professional organizations, and health-related websites) and personal narratives of non–health care professionals (patients, independent users). The videos were independently analyzed by 2 raters (YHP and REC) and coded according to the themes of “Educational content” and “Personal narratives.” Discrepancies in coding were resolved through repetitive discussions and consensus, ensuring a reliable and consistent categorization process. This method of assessment has been used in similar studies of other diseases [10].

### Video Usefulness

The usefulness score is a usefulness scale defined by Lee et al [11]. Each video is rated with a score between 0 and 10 depending on the content of the video, such as causes, symptoms, diagnosis, diagnosis, and recovery status. According to the total score obtained, it is categorized as follows: 0=not useful, 1-3=less useful, 4-7=useful, and 8-10=very useful.

### Video Popularity

The video power index (VPI) developed by Erdem et al [12] shows the popularity of videos and has been used in many studies [9]. The VPI calculation is as follows: VPI=(×100/ [number of likes+number of dislikes]) × (number of views/number of d since upload)/100.

### Quality and Reliability Evaluation

The Global Quality Scale (GQS) assesses the quality by providing the interpretation and usefulness of the videos for patients based on the flow of information. GQS has a 5-point Likert structure according to the quality, flow, and ease of use of the analyzed videos [13]. As used in similar studies, scores 1-2 were considered as low quality (inadequate in terms of patient information, contains incomplete information), 3 as medium quality (video flow is poor, some information is available but important issues are not addressed), and 4-5 (contains sufficient and useful information for patients) as high quality [14].

The quality assessment included the *Journal of the American Medical Association* (*JAMA*) benchmark criteria for determining authorship, attribution, disclosure, and currency. Each of these criteria was given a score of 1, with a maximum score of 4 [15].

The mDISCERN scale developed by Charnock et al [16] and later adapted to YouTube videos by Singh et al [17] was used to assess the reliability of the videos. The mDISCERN scale consists of 5 questions and is a questionnaire about information sources, purpose, reliability, bias, additional sources, and areas of uncertainty. Each question can be answered yes or no. Each yes answer is worth 1 point and 5 points represent the highest quality.

The video content was evaluated and graded according to the most recent American College of Gastroenterology guidelines for the management of CD [18]. These guidelines emphasize accurate symptom identification, diagnostic criteria, and effective dietary management strategies. Videos were scored for reliability, usefulness, and consistency with evidence-based practice.

### Statistical Analyses

The SPSS (version 25.0 for Windows; IBM Corp) package program was used. Continuous variables were evaluated using the Shapiro-Wilk test to determine whether they were normally distributed. Continuous variables are reported as median and IQR, while categorical variables are presented as counts and percentages. Chi-square tests were used to analyze categorical variables and Mann-Whitney *U* test for numerical variables. The significance level was set at *P*=.05 for all analyses.

### Ethical Considerations

The study adhered to the ethical standards outlined in the Helsinki Declaration and complied with national regulations in the respective field. Since the study did not involve the use of human or animal data, ethics committee approval was not necessary. This study analyzed publicly available YouTube videos. No identifiable personal data was used, and all results are presented in aggregate. Therefore, formal ethics approval was not required.

## Results

### Main Characteristics of Videos and Video Analysis

In total, 120 videos were analyzed and 85 videos met the study criteria and were included. A total of 35 videos were excluded from the study, including 2 non-English language videos, 13 videos with repetitive content, 12 videos with advertising content, and 8 videos with poor picture and sound quality. Most (22/85, 25.9%) were published by universities and other organizations, and most (50/85, 59%) were uploaded by health care professionals. A total of 68.2% (58/85) of the videos consisted of real images. Descriptive statistics of the above characteristics and other variables are shown in Table 1.

**Table 1:** Main characteristics of the analyzed videos. Categorical variables are expressed as n (%), and numerical variables are expressed as median (Q1-Q3).

Characteristics			Values
**Source, n (%)**			
		Physicians	12 (14
		Universities and professional organizations	22 (26)
		Health information websites	16 (19)
		Independent users	16 (19)
		Patient	19 (22)
**Source, n (%)**			
		Health care professionals	50 (59)
		Non–health care professionals	35 (41)
**Image type**			
		Real image, n (%)	58 (68)
		Animation, n (%)	27 (32)
		Number of views, median (IQR)	17,026 (2860-46,358)
		Number of likes, median (IQR)	306 (45-820)
		Number of dislikes, median (IQR)	6 (1-20)
		Duration (min), median (IQR)	6.3 (3.4-12.1)
		Days on YouTube, median (IQR)	1381 (572-2290)
		Number of comments, median (IQR)	27 (5-130)
		Views per day, median (IQR)	13.1 (4-33.2)
		Likes per day, median (IQR)	0.2 (0.1-0.7)

### Content Analysis and Source Evaluation of Videos

In the health care professional group, most (37/85, 43.1%) of the videos were uploaded by universities and other organizations, whereas in the non–health care professional group, most (19/34, 55.9%) of the videos were uploaded by “patients” (*P*<.001). While the duration of the videos uploaded by health care professionals was significantly longer than the other group (*P*=.009), it was concluded that the median scores for mDISCERN (4, IQR 4-5 vs 2, IQR 2-3; *P*<.01), GQS (4, IQR 4-5 vs 3, IQR 2-3; *P*<.001), *JAMA* (4, IQR 3-4 vs 2, IQR 2-3; *P*<.001), and usefulness (8, IQR 7-9 vs 6, IQR 3-6; *P*<.001) of the videos from this group were significantly higher than those from non–health care professionals. (Tables 2 and 3)

**Table 2:** The average scales of the analyzed videos.

Video scales	Values, median (IQR)
mDISCERN^a^	3 (3-4)
GQS^b^	4 (3-4)
*JAMA* ^c^	3 (2-4)
VPI^d^	12.8 (4-33)
Usefulness	7 (5-9)

^a^mDISCERN: modified DISCERN score.

^b^GQS: Global Quality Scale score.

^c^*JAMA*: Journal of the American Medical Association.

^d^VPI: video power index.

**Table 3:** Comparison of videos according to source status. Categorical variables are expressed as n (%), and numerical variables as median (Q1-Q3).

Variables			Source				*P* value
			Health care professionals		Non–health care professionals		
**Image**							
	Real image, n (%)	31 (62)		27 (77.1)		.21	
	Animation, n (%)	19 (38)		8 (22.9)			
	Number of views, median (IQR)	16,657 (4858-57,896)		17,851.5 (1907-43,310)		.87	
	Number of likes, median (IQR)	297 (52-774)		373 (22-846)		.67	
	Number of dislikes, median (IQR)	6 (1-24)		8.5 (0-18)		.92	
	Duration (min), median (IQR)	7.4 (4.2-16.4)		3.9 (2.5-8.2)		.009	
	Days on YouTube, median (IQR)	1291 (516-2290)		1467.5 (832-2470)		.64	
	Number of comments, median (IQR)	21 (6-79)		67 (3-170)		.52	
	View per day, median (IQR)	12.8 (4.6-40.9)		15.6 (2.1-33.2)		.50	
	Like per day, median (IQR)	0.23 (0.07-1)		0.18 (0.03-0.73)		.39	
	mDISCERN^a^, median (IQR)	4 (4-5)		2 (2-3)		<.001	
	GQS^b^, median (IQR)	4 (4-5)		3 (2-3)		<.001	
	*JAMA^c^*, median (IQR)	4 (3-4)		2 (2-2)		<.001	
	VPI^d^, median (IQR)	12.3 (4.6-41)		15.3 (2.1-33)		.72	
	Usefulness, median (IQR)	8 (7-9)		5 (3-6)		<.001	

^a^mDISCERN: modified DISCERN score.

^b^GQS: Global Quality Scale score.

^c^*JAMA: Journal of the American Medical Association*.

^d^VPI: video power index.

### Themes Identified in Videos

From the 85 included videos, two major themes were identified.

#### Educational Content

These videos, primarily created by health care professionals, provided detailed information about CD symptoms, diagnosis, treatment, and long-term management. This category accounted for 59% (50/85) of all videos and demonstrated significantly higher scores in quality and reliability metrics (mDISCERN, GQS, *JAMA*, and Usefulness; *P*<.001).

#### Personal Narratives

Uploaded by patients or non–health care professionals, these videos focused on personal journeys, sharing challenges, and tips for living with CD. They received moderate interaction metrics (likes, comments) but were lower in quality and reliability scores (*P*<.001).

## Discussion

### Principal Findings

In this study, we analyzed YouTube videos about CD, an important disease that can occur at any age. We found that CD videos uploaded by health care professionals were significantly more reliable, adequate, useful, and quality information sources than those uploaded by non–health care professionals. Another striking result of the study was that there was no difference in video interaction parameters between those with and without health care professionals as video sources.

Recently, SM has become a popular way to access medical information and knowledge. Patients with many chronic diseases, including CD, have been reported to use SM as a source of information since adolescence [19]. Especially YouTube, a video sharing website, has become an important source of information in the field of health. In a recent nationally based survey study, it was reported that younger patient groups and patients with chronic diseases such as hypertension, diabetes mellitus, and chronic lung disease were more likely to watch YouTube videos as a source of health-related information [20].

As in other chronic diseases, SM use among patients with CD and their families has become widespread in recent years [4]. When we consider the importance of increasing adherence to a gluten-free diet as well as the diagnosis, risk factors, and clinical presentation of the disease, access to real and adequate information through SM becomes even more important. In a recent survey of patients with CD, two-thirds of the patients used SM every day for an average of 60 minutes per day. The 3 most common reasons for using SM were researching gluten-free diet products, obtaining information about diet, and CD. In the study, it was stated that the most frequently used platform was WhatsApp (Meta), and it was suggested that YouTube usage was 4% [4]. Although this rate may vary according to regional and cultural differences, it is still a relatively low rate and suggests that the use of YouTube may be higher than this data. In another similar survey study conducted in Japan, 27% of more than 2000 participants with chronic diseases stated that they used the YouTube platform related to their disease [20].

One of the studies evaluating YouTube videos on CD was a study in which 100 videos were evaluated in 2019. In this study, it was examined whether there was a difference between sources in 31 different topics such as etiology, symptoms, diagnosis, and treatment of the disease, and it was stated that there was no significant difference in terms of content in all remaining topics except 3 [7]. However, none of the video reliability-efficacy tests used in our study were used in this study. Nevertheless, it differs from our study because it claims that there is mostly no significant difference between videos whose source is health care professionals and other videos in terms of topics. Another study in the literature evaluated Polish-language videos, so it does not seem possible to make a comparison with our study [8].

Among the videos analyzed in our study, the fact that the reliability, usefulness, and quality scores of the videos of health care professionals were significantly higher than those of non–health care professionals was also observed in similar studies evaluating other diseases [21]. One of the most remarkable findings of our study is that there was no significant difference between the groups in terms of views, likes, dislikes, and VPI. There are many factors that can contribute to this, such as the visual presentation of the video, the demographic and cultural make-up of the viewers, the video’s viral status, and the influencer’s effect [22,23]. In a recent study investigating the influencer effect on SM related to dermatology, it was shown that dermatologists without competence and certification had as high a level of interaction as those with competence and certification [23]. This finding shows us that videos that may be insufficient as a source of information may also have high interaction and accordingly may cause misinformation and negative effects on patients and their families.

Based on these findings, we believe that in order for YouTube to be an accurate source of information about CD, many organizations and institutions, such as professional associations and universities, should provide training for health care professionals to produce high-quality videos that can provide more interaction and raise awareness among health care professionals about this issue. On the other hand, it is also important to raise patient awareness of the possibility that patients may be exposed to misinformation when using YouTube. We think that more use of YouTube and other SM platforms by health care professionals and peer review of health-related video content may reduce misinformation.

### Limitations

There were some limitations in our study. The first 120 videos searched with the keyword “Celiac disease'” in the search results were analyzed and the other videos were not analyzed. In addition, since YouTube is a dynamic SM platform, video interaction parameters such as daily views, likes, and dislikes can change every day. Finally, the fact that only English videos were analyzed in our study can be considered among the limitations.

### Conclusions

This study showed that YouTube videos about CD vary significantly in reliability and quality depending on their source. Increasing the production of reliable videos by health care professionals may help to improve patient education and make YouTube a more reliable resource.

## Abbreviations

CD: celiac disease
GQS: Global Quality Scale
*JAMA*: *Journal of the American Medical Association*
mDISCERN: modified DISCERN
SM: social media
VPI: video power index
